# Investigation of Macular Structural and Microcirculatory Characteristics of Posterior Staphyloma in High Myopic Eyes by Swept Source Optical Coherence Tomography Angiography

**DOI:** 10.3389/fphys.2022.856507

**Published:** 2022-04-07

**Authors:** Haoru Li, Qingxin Wang, Yucheng Liu, Xin Wang, Qing He, Yanhui Chen, Ruihua Wei

**Affiliations:** Tianjin Key Laboratory of Retinal Functions and Diseases, Tianjin Branch of National Clinical Research Center for Ocular Disease, Eye Institute and School of Optometry, Tianjin Medical University Eye Hospital, Tianjin, China

**Keywords:** high myopia, posterior staphyloma, microcirculation, OCT angiography, macular structures

## Abstract

**Purpose:** To investigate the characteristics of macular structures and microcirculation of posterior staphyloma (PS) and explored factors related to PS in eyes with high myopia.

**Methods:** There were 114 eyes of 82 patients in this study. Using 1:1 matching of the axial length of myopic eyes, patients were divided into no PS (NPS) and PS groups. Comprehensive ophthalmic examinations were performed on all patients. Structural parameters were acquired using swept source optical coherence tomography (SS-OCT). OCT angiography (OCTA) was used to measure the microcirculation parameters. Generalized estimated equation and linear correlation analysis were used for the statistical analysis.

**Results:** Patients with PS had a thinner retinal thickness (RT) and choroid thickness (CT) (all *p* < 0.05) in the measurement areas and a significantly lower subfoveal scleral thickness (SFST) (*p* < 0.001) than those without PS. Retinal deep vascular complex density (DVD) (all *p* < 0.05) and choriocapillaris perfusion area (CCPA) (all *p* < 0.001) were significantly lower in the measurement areas of the PS group than in those of the NPS group. There was no significant difference in the retinal superficial vascular density between the two groups. Generalized estimating equation indicated that SFST (*B* = 0.079, *p* = 0.001), parafoveal RT (B = −0.162, *p* = 0.041), foveal CT (*B* = 0.292, *p* = 0.013), parafoveal CT (*B* = −0.157, *p* = 0.023), foveal CCPA (*B* = 0.691, *p* = 0.003) and parafoveal CCPA (*B* = −0.026, *p* = 0.004) were significantly correlated with PS. Age (*r* = −0.323, *p* = 0.001), spherical equivalent refraction (SER) (*r* = 0.289, *p* = 0.004), subfoveal CT (*r* = 0.398, *p* < 0.001), foveal DVD (*r* = 0.346, *p* < 0.001), foveal CT (*r* = 0.429, *p* < 0.001), and foveal CCPA (*r* = 0.387, *p* < 0.001) were strongly correlated with SFST.

**Conclusions:** The macular structures and microcirculation in the PS group were different from those in the NPS group. SFST, CT, and CCPA were significantly correlated with PS. Lower SFST in PS was correlated with abnormalities of CT and microcirculation.

## Introduction

The prevalence of high myopia is continuously increasing worldwide, especially in East Asian countries, and pathological myopia may cause irreversible visual damage (Iwase et al., 2006; Xu et al., 2006; Chen et al., 2012; Varma et al., 2016). It is predicted that by 2050, there will be 938 million people (about 9.8% of the world population) with high myopia (Holden et al., 2016). High myopia is often accompanied by potentially pathological changes, such as retinal detachment, retinoschisis, or posterior staphyloma (PS), which seriously affect visual acuity and cannot be prevented or treated by optical correction (Ohno-Matsui et al., 2016). PS is a common pathological alteration in highly myopic eyes and a typical sign of pathological myopia. It has been defined as ectasia of a limited portion of the eyeball (Ohno-Matsui, 2014). The appearance and progression of PS are also frequently concurrent with other macular lesions (Shinohara et al., 2018; Mimura et al., 2019), causing an increased risk of blindness.

The sclera has been considered as the major tissue in the formation of PS. However, it is gradually being indicated that the choroid or other tissues may also be initiating factors for the occurrence and development of PS (Jonas and Panda-Jonas, 2016; Ohno-Matsui and Jonas, 2019). The etiology of PS remains unknown, and its pathogenesis remains controversial. Current studies have shown that there are differences in structures and microcirculation between eyes with different degrees of myopia (La Spina et al., 2016; Al-Sheikh et al., 2017; Zhou et al., 2018); however, the observation and analysis of PS still require detailed studies. Reduced scleral thickness and choroidal thickness have also been reported in PS (Tanaka et al., 2019; Park et al., 2021). Park et al. (2021) first quantified the decrease in scleral thickness *in vivo*. Structural abnormalities inevitably lead to changes in blood circulation, yet the features of microcirculation in PS and their correlation are still unclear at present. Alterations in the structure or function of the macula may affect the function of photoreceptor cells in the fovea and impair the transmission of visual signals. SS-OCT/OCTA is an advanced machine equipped with the characteristics of rapidity, non-invasion, and strong repeatability. It turns two-dimensional blood flow images into more intuitive, quantifiable, and calculable parameters (Spaide et al., 2018), which provided technical support for our research.

Therefore, this study aimed to investigate the characteristics of macular structures and microcirculation with PS under the premise of 1:1 matching of axial length (AL). Unlike most studies, we matched AL to reduce its interference with the study results. Second, the factors which were significantly correlated with PS in eyes with high myopia were explored. Third, we also investigated the correlation between scleral thickness (ST) and various ocular parameters in eyes with high myopia, to provide a basis for possible pathological mechanisms of PS and macular lesions.

## Materials and Methods

### Subjects

This was a cross-sectional study. A total of 82 patients were recruited from Tianjin Medical University Eye Hospital between December 2020 and October 2021. All study procedures adhered to the tenets of the Declaration of Helsinki and were approved by the ethics committee of Tianjin Medical University Eye Hospital. The initial inclusion criteria were age >18 years, intraocular pressure (IOP) between 10 and 21 mmHg (1 mmHg = 0.133 kPa), spherical equivalent refraction (SER) ≤ −6.00 D, and AL ≥ 26 mm. Patients with the following criteria were excluded: patients with systemic or ocular diseases, such as glaucoma, diabetes, or hypertension; retinal diseases; myopic pathological changes, such as macular hole, retinal detachment, choroidal neovascularization, severe retinal choroidal atrophy, or refractive stromal opacity.

### Ophthalmic Examinations

**FIGURE 1:**
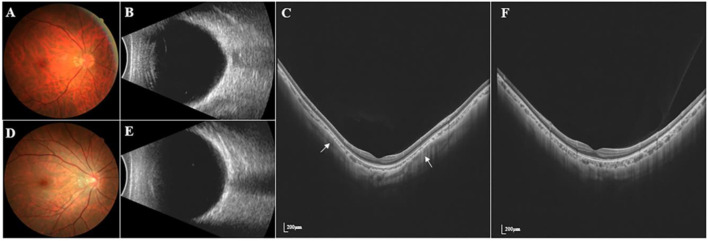
Fundus photography, B-ultrasound and SS-OCT images of PS and NPS. **(A–C)** Right eye images of a 39-year-old male with PS, whose AL was 28.96 mm. **(D–F)** Right eye images of a 28-year-old male without PS, whose AL was 28.66 mm. The white arrow points to the edge of the PS. The thickness of the choroid was the thinnest at the edge of the staphyloma, being thickened gradually from the edge to the periphery and the posterior pole of the fundus.

All patients underwent a complete ophthalmic examination, including slit-lamp biomicroscopy, IOP (CT-1; Topcon, Japan), free mydriatic fundus photography (CR-2, Canon, Japan), B-ultrasound (MD-2300S, Maida, China), best-corrected visual acuity (BCVA), SER (KR-800, Topcon, Japan), SS-OCT/OCTA (VG200S, SVision Imaging, Henan, China), and AL (Lenstar LS-900, Haag-Streit AG, Switzerland). According to the presence or absence of PS and matched for the AL (±0.3 mm), 114 eyes were assigned to either the no PS (NPS) group (n = 57) or PS group (n = 57). An example of two eyes with matched AL is shown in Figure 1.

### SS-OCT/OCTA Image Acquisition

The SS-OCT/OCTA system we used in this study was equipped with an eye tracking device which eliminated eye movement artifacts. In addition, the system contained an SS laser with a central wavelength of approximately 1,050 nm and a scan rate of 200,000 A-scans per second (Wu et al., 2021a). At the same time, the scan size was adjusted according to the differences in magnification due to different ALs of the eyes, which was its most distinctive and advanced feature.

**FIGURE 2:**
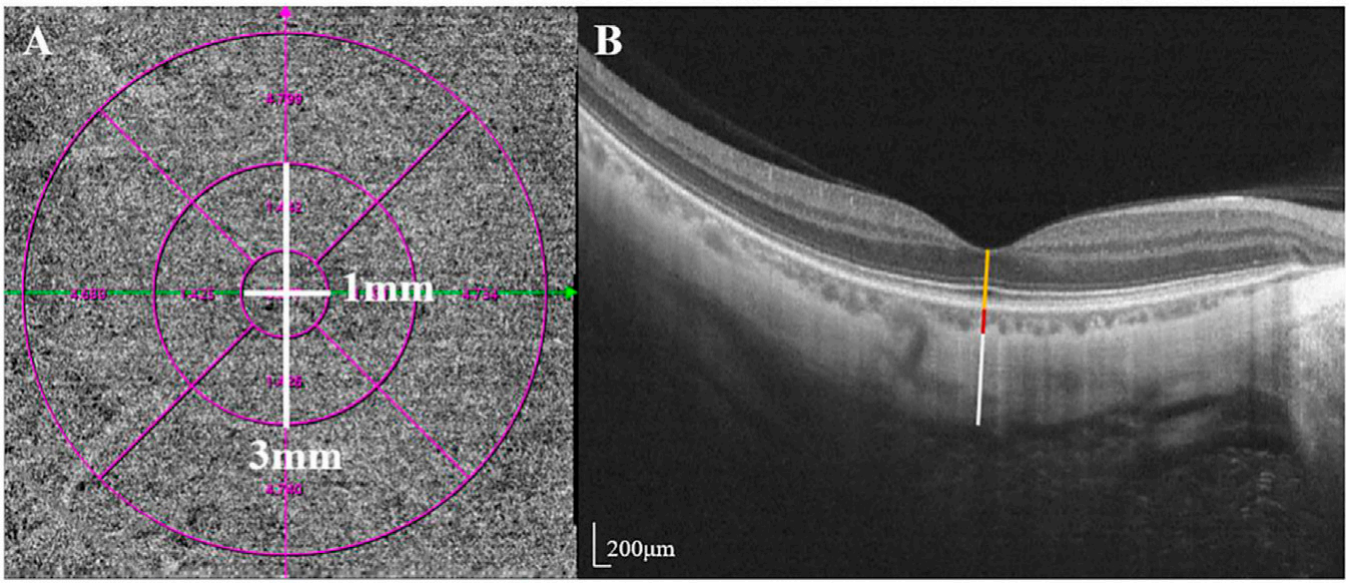
Two areas of macular and manually measured parameters (RT, CT, and ST). **(A)** The foveal region was a circular area with a diameter of 1 mm, centered on the fovea. The parafoveal region was a ring area around the fovea with an inner diameter of 1 mm and an outer diameter of 3 mm **(B)** Measurement of SFRT (yellow line), SFCT (red line) and SFST (white line). RT was defined as the vertical distance between the inner limiting membrane and the retinal pigment epithelium. CT was defined as the vertical distance between the outer edge of the retinal pigment epithelium (RPE) and the outer edge of the choroid. ST was defined as the vertical distance between the outer edge of the choroid and the outer border of the sclera.

**FIGURE 3:**
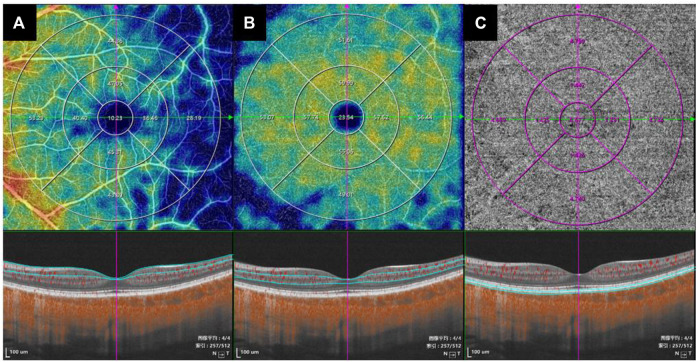
The value of the microcirculation parameters were calculated automatically by the system. The top en-face images are intensity projections of the regions selected by the blue lines in the bottom images. **(A)** The retinal superficial vascular complex was automatically defined by the system as microvessels found 5 μm above the inner limiting membrane to one-third interface of ganglion cell layer and inner plexiform layer (GCL + IPL). **(B)** The retinal deep vascular complex was automatically defined by the system as one-third interface of GCL + IPL to 25 μm below the lower border of the inner nuclear layer. **(C)** The choriocapillaris was automatically defined by the system as the microvasculature from the basal border of the RPE-Bruch’s membrane complex to 20 μm below it.

SS-OCT/OCTA measurements were performed with 36 radial scans with a length of 16 mm and a depth of 6 mm centered on the fovea. Cube scans of 6 mm × 6 mm were centered on the fovea. The macular area was divided into foveal and parafoveal regions. The foveal region was a circular area with a diameter of 1 mm, centered on the fovea. The parafoveal region was a ring area around the fovea with an inner diameter of 1 mm and an outer diameter of 3 mm (Figure 2A). The major automation outcomes recorded included subfoveal retinal thickness (SFRT), subfoveal choroid thickness (SFCT), subfoveal scleral thickness (SFST), and retinal thickness (RT), choroid thickness (CT), retinal superficial vascular complex density (SVD), retinal deep vascular complex density (DVD), and choriocapillaris perfusion area (CCPA). RT was defined as the vertical distance between the internal limiting membrane and the retinal pigment epithelium. CT was defined as the vertical distance between the outer edge of the retinal pigment epithelium (RPE) and the outer edge of the choroid. ST was defined as the vertical distance between the outer edge of the choroid and the outer border of the sclera. SFRT, SFCT, and SFST were measured manually and averaged by two experienced ophthalmologists. The manually measured parameters are shown in Figure 2B. The retinal superficial vascular complex was automatically defined by the system as microvessels found 5 μm above the inner limiting membrane to one-third interface of ganglion cell layer and inner plexiform layer (GCL + IPL). The retinal deep vascular complex was automatically defined by the system as one-third interface of GCL + IPL to 25 μm below the lower border of the inner nuclear layer. The choriocapillaris was automatically defined by the system as the microvasculature from the basal border of the RPE-Bruch’s membrane complex to 20 μm below it (Campbell et al., 2017; Wu et al., 2021b) (Figure 3).

### Diagnosis of Posterior Staphyloma

We assessed 36 radial scans using SS-OCT. The presence of PS was determined using typical SS-OCT features. In this study, the diagnosis of PS was based on the following criteria: the thickness of the choroid was the thinnest at the edge of the staphyloma, being thickened gradually from the edge to the periphery and the posterior pole of the fundus (Shinohara et al., 2017; Liu et al., 2021). PS was judged to be present when the criteria mentioned above were observed in a minimum of six consecutive cross-sections in the 36 radial scans. All image evaluations were performed by the same examiner, with the basic patient information masked. Questionable images were determined by an experienced ophthalmology team. We only enroll both eyes when there was PS on both eyes. Each eye being paired with a different patient AL-matching.

### Statistical Analysis

Statistical analyses were performed using SPSS software (version 26.0, SPSS, IBM, Chicago). The normality of the data was tested using the Kolmogorov-Smirnov normality test. Chi-square test, Independent *t*-test, and Mann-Whitney *U* test were used to assess differences between the two groups as appropriate. Continuous variables were expressed as mean ± standard deviation. Generalized estimating equation (GEE) was used to identify the independent variables correlated with PS. Linear correlation analysis was used to explore the correlation between SFST and ocular parameters in eyes with high myopia. Spearman’s or Pearson’s correlation coefficients were used to indicate the correlation between SFST and ocular parameters, as appropriate. Statistical significance was set at *p* < 0.05.

## Results

### Baseline Measurements

A total of 114 eyes from 82 patients with high myopia who met the study inclusion criteria were analyzed. There were 57 eyes in PS group, and 57 eyes in NPS group. The mean age of the subjects was 32.46 ± 11.29 years old, the mean BCVA (logMAR) was 0.05 ± 0.11, the mean SER was −10.15 ± 2.96 D, the mean AL was 27.67 ± 0.83 mm. The age of patients with PS (35.19 ± 12.27) was older than those without PS (29.74 ± 9.56) (*p* = 0.015). There were no statistical differences in Gender, IOP, BCVA, SER, and AL between the two groups (all *p* > 0.05) (Table 1).

**TABLE 1:** Demographics and baseline characteristics of NPS group and PS group.

Parameters	NPS Group (n = 57)	PS Group (n = 57)	Test Value	*p* Value
Age	29.74 ± 9.56	35.19 ± 12.27	Z = −2.442	**0.015** ^**‡**^
Gender (male/female)	22/35	15/42	χ2 = 1.961	0.161^*^
Iop (mmHg)	15.92 ± 2.52	16.05 ± 2.67	Z = −0.233	0.816^**†**^
BCVA (logMAR)	0.04 ± 0.08	0.06 ± 0.14	Z = −1.499	0.134^**†**^
SER (D)	−9.81 ± 3.09	−10.48 ± 2.81	Z = −1.629	0.103^**†**^
AL (mm)	27.64 ± 0.86	27.70 ± 0.82	t = −0.404	0.687^**‡**^

*BCVA*, best-corrected visual acuity; *SER*, spherical equivalent refraction; *AL*, axial length. * Chi-square test. † Independent *t*-test. ‡ Mann-Whitney U test. Parameters with statistical significance are shown in boldface.

### Macular Structures and Microcirculation Parameters of PS Group and NPS Group

The SFRT (*p* = 0.021) and SFCT (*p* < 0.001) in PS group were lower than those in NPS group. The SFST of 10 eyes in the NPS group and six eyes in the PS group were difficult to measure. Therefore, the SFST of 47 eyes in the NPS group was compared with 51 eyes in the PS group. The results showed that SFST in the PS group was significantly lower than that in the NPS group (*p* < 0.001). Moreover, foveal RT (*p* = 0.033), parafoveal RT (*p =* 0.013), foveal CT (*p* < 0.001), and parafoveal CT (*p* < 0.001) in the PS group were all lower than those in the NPS group (Table 2). Eyes with PS had a significantly lower foveal and parafoveal DVD (all *p* < 0.05) and a lower foveal and parafoveal CCPA (both *p* < 0.001) compared to those without PS. There were no significant differences in foveal and parafoveal SVD (*p* > 0.05) between the PS and NPS groups (Table 3).

**TABLE 2:** Comparison of RT, CT, and SFST in NPS group and PS group.

Parameters	NPS Group (n = 57)	PS Group (n = 57)	Test Value	*p* Value
SFRT(μm)	216.76 ± 24.89	205.24 ± 26.50	Z = −2.315	**0.021** ^‡^
SFCT (μm)	203.96 ± 69.17	146.44 ± 77.73	Z = −4.406	<**0.001** ^‡^
SFST (μm)	405.60 ± 60.49 (n = 47)	310.39 ± 63.33 (n = 51)	t = 6.836	<**0.001** ^†^
RT (μm)				
foveal	254.50 ± 21.48	246.52 ± 19.40	Z = −2.134	**0.033** ^‡^
parafoveal	324.91 ± 16.79	317.92 ± 17.60	Z = −2.485	**0.013** ^‡^
CT (μm)				
foveal	239.00 ± 66.60	150.31 ± 49.52	t = 8.068	<**0.001** ^†^
parafoveal	237.27 ± 59.54	176.22 ± 68.23	Z = −4.871	<**0.001** ^‡^

*SFRT*, subfoveal retinal thickness; *SFCT*, subfoveal choroid thickness; *SFST*, subfoveal scleral thickness; *RT*, retinal thickness; *CT*, choroid thickness. † Independent *t*-test. ‡ Mann-Whitney U test. Parameters with statistical significance are shown in boldface.

**TABLE 3:** Comparison of SVD, DVD, and CCPA of NPS group and PS group.

Parameters	NPS Group (n = 57)	PS Group (n = 57)	Test Value	*p* Value
SVD (%)				
foveal	8.03 ± 3.05	8.08 ± 3.22	Z = −0.173	0.863^‡^
parafoveal	41.45 ± 5.22	41.31 ± 5.24	Z = −0.252	0.801^‡^
DVD (%)				
foveal	20.97 ± 6.00	16.92 ± 6.71	t = 3.395	**0.001** ^†^
parafoveal	54.70 ± 3.80	51.66 ± 6.05	Z = −3.103	**0.002** ^‡^
CCPA(mm^2^)				
foveal	0.70 ± 0.05	0.60 ± 0.11	Z = −6.081	<**0.001** ^‡^
parafoveal	5.37 ± 0.51	4.91 ± 0.67	Z = −4.146	<**0.001** ^‡^

*SVD*, superficial retinal vascular density; *DVD*, deep retinal vascular density; *CCPA*, choriocapillaris perfusion area. † Independent *t*-test. ‡ Mann-Whitney U test. Parameters with statistical significance are shown in boldface.

### The Factors Significantly Correlated With PS

The GEE revealed the relationship between PS, macular structures, and microcirculation parameters. The GEE demonstrated that SFST (*B* = 0.079, *p* = 0.001), parafoveal RT (*B* = −0.162, *p* = 0.041), foveal CT (*B* = 0.292, *p* = 0.013), parafoveal CT (*B* = −0.157, *p* = 0.023), foveal CCPA (*B* = 0.691, *p* = 0.003) and parafoveal CCPA (*B* = −0.026, *p* = 0.004) were correlated with PS (Table 4).

**TABLE 4:** Analysis of correlations between PS and variables by generalized estimating equation.

Parameters	*B* Value	Standard Error	Wald Value	OR	95%CI	*p* Value
age	0.143	0.081	3.125	1.153	0.985–1.351	0.077
SFRT(μm)	0.139	0.099	1.950	1.149	0.946–1.395	0.163
SFCT (μm)	−0.047	0.040	1.376	0.954	0.883–1.032	0.241
SFST (μm)	0.079	0.023	11.715	1.082	1.034–1.133	**0.001**
foveal RT (μm)	−0.023	0.115	0.042	0.977	0.780–1.223	0.838
parafoveal RT (μm)	−0.162	0.079	4.179	0.850	0.728–0.993	**0.041**
foveal CT (μm)	0.292	0.118	6.122	1.339	1.063–1.687	**0.013**
parafoveal CT (μm)	−0.157	0.069	5.149	0.854	0.746–0.979	**0.023**
foveal DVD (%)	0.085	0.112	0.571	1.088	0.874–1.355	0.450
parafoveal DVD (%)	−0.112	0.166	0.456	0.894	0.646–1.237	0.499
foveal CCPA(mm^2^)*100	0.691	0.236	8.595	1.996	1.257–3.168	**0.003**
parafoveal CCPA(mm^2^)*100	−0.026	0.009	8.517	0.975	0.958–0.992	**0.004**

*B* unstandardized coefficient. *SFRT*, subfoveal retinal thickness; *SFCT*, subfoveal choroid thickness; *SFST*, subfoveal scleral thickness; *RT*, retinal thickness; *CT*, choroid thickness; *DVD*, deep retinal vascular density; *CCPA*, choriocapillaris perfusion area. Parameters with statistical significance are shown in boldface.

### Correlation Between SFST and Various Ocular Parameters

**FIGURE 4:**
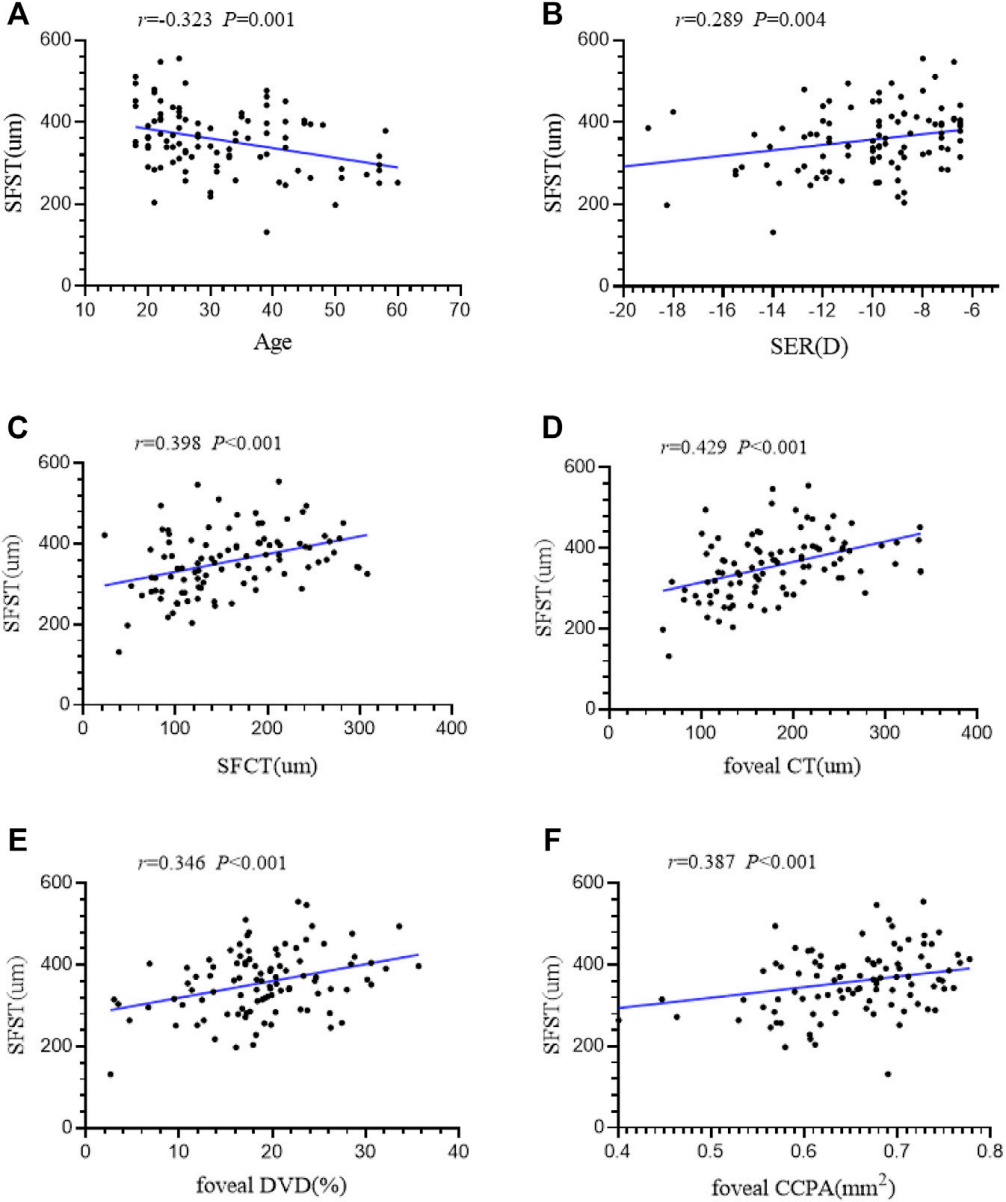
(**A–F)** The correlation between SFST and age, SER, SFCT, foveal CT, foveal DVD and foveal CCPA in 98 high myopic eyes were demonstrated. *AL* axial length, *SER* spherical equivalent refraction, *SFRT* subfoveal retinal thickness, *SFCT* subfoveal choroid thickness, *SFST* subfoveal scleral thickness, *SVD* superficial retinal vascular density, *DVD* deep retinal vascular density, *CCPA* choriocapillaris perfusion area.

Linear correlation analysis was used to explore the correlation between SFST and ocular parameters in eyes with high myopia. We found that SFST was significantly correlated with age (*r* = −0.323, *p* = 0.001). In people with high myopia, the older they were, the thinner the SFST was. In addition, SFST was significantly correlated with SER (*r* = 0.289, *p* = 0.004), SFCT (*r* = 0.398, *p* < 0.001), foveal CT (*r* = 0.429, *p* < 0.001), foveal DVD (*r* = 0.346, *p* < 0.001), and foveal CCPA (*r* = 0.387, *p* < 0.001). SFST was lower in eyes with lower SER, lower SFCT and foveal CT, lower foveal DVD, and foveal CCPA (Figure 4). AL, SFRT, foveal RT, and foveal SVD were not correlated with SFST (all *p* > 0.05) (Table 5).

**TABLE 5:** Linear correlation analysis between SFST and ocular variables.

	SFST	
	*r* value	*p* value
age	−0.323	**0.001**
AL (mm)	−0.136	0.182
SER(D)	0.289	**0.004**
SFRT(μm)	0.124	0.224
SFCT (μm)	0.398	<**0.001**
foveal RT (μm)	0.123	0.226
foveal CT (μm)	0.429	<**0.001**
foveal SVD (%)	0.004	0.966
foveal DVD (%)	0.346	<**0.001**
foveal CCPA(mm^2^)	0.387	<**0.001**

Linear correlation analysis between SFST, and ocular parameters; *AL*, axial length; *SER*, spherical equivalent refraction; *SFRT*, subfoveal retinal thickness; *SFCT*, subfoveal choroid thickness; *SFST*, subfoveal scleral thickness; *SVD*, superficial retinal vascular density; *DVD*, deep retinal vascular density; *CCPA*, choriocapillaris perfusion area. Parameters with statistical significance are shown in boldface.

## Discussion

The purpose of this study was to observe the characteristics of macular structures and microcirculation in AL-matched highly myopic eyes with PS. The factors correlated with PS in eyes with high myopia were explored. This study provides a new perspective for exploring the pathological mechanism of PS.

In this study, patients in PS group were older than NPS group (35.19 ± 12.27 vs. 29.74 ± 9.56 years). This indicated that age was probably an influencing factor for the presence of PS, as reported in a previous study (Ohno-Matsui and Jonas, 2019). However, GEE in this study did not show that age was correlated with PS. This was probably due to the differences in sample size and the matching of the AL that caused this result. It has been shown that aging affects the fundus microcirculation, and the choroidal angioarchitecture distinctly declines from 40 years of age (Adhi et al., 2015; Nivison-Smith et al., 2020). The mean age of patients in the two groups of this study was less than 40 years old. Thus we think this can minimize the deviation due to age, and may be considered to have no significant influence on our results.

The machine used in this study was equipped with 36 radial scans with a length of 16 mm and a depth of 6 mm scanning range, as well as high-quality images. This enabled us to measure the RT, CT, and especially SFST. The RT, CT, and SFST in the macular area of the PS group were lower than those in the NPS group, and GEE indicated that SFST and foveal and parafoveal CT were significantly correlated with PS. This result was consistent with many previous reports (Hayashi et al., 2013; Igarashi-Yokoi et al., 2021; Nie et al., 2021). Zhou et al. also found a significant correlation between PS and choroidal thinning; the higher the relative height of PS, the more serious the choroidal atrophy (Zhou et al., 2017). A study based on CT and ST in 237 eyes with PS revealed that compared with the NPS group, while AL in the PS group was significantly increased, CT and ST at each measurement point were decreased (Park et al., 2021). However, RT was not described in that study. In the current study, parafoveal RT related to the presence of PS. The decrease in RT of the macular area can be explained by the elongation of the AL. As the AL lengthens, the choroid and sclera were also stretched axially. The choroid was thinnest at the edge of the PS (Shinohara et al., 2017), and the extremely thin retina and choroid were less resistant to intraocular pressure and axial stretching. This resulted in uneven stress of the adjacent sclera and surrounding tissue, and subsequently, a partial abnormal bulging of the eyeball. Uneven atrophy and thinning of the choroid may be the reason for the change in scleral curvature (Tanaka et al., 2019). Sudden changes in the scleral curvature may lead to abnormalities in the microvascular structure of the corresponding area, which result in alteration of the microcirculation and damaged retinal function, aggravated visual loss, and even deteriorated normal visual function.

DVD and CCPA in the macular area of the PS group were lower than NPS group. Nie et al. (2021) also indicated that there were significant differences in foveal DVD and macular CCPA between the PS and NPS groups; however, this phenomenon was not observed in the parafoveal DVD. The results of our study showed that the DVD of the parafoveal in the PS group was also lower than that in the NPS group, which may be due to the differences in the samples we included. Whether the location and extent of PS involvement causes differences in the parafoveal DVD requires further investigation. A previous study has shown that SVD and DVD in the macular area decreased with the stretching of AL (Yang et al., 2020). Furthermore, differences in image magnification due to different ALs will affect vascular density (Sampson et al., 2017; Fu et al., 2021). The scanning range of the SS-OCT/OCTA we applied can correct the difference in fundus magnification due to the different Als, to reduce the intervention of AL on the results of this study. Fan et al. (2017) and Yang et al. (2020) found that with the growth of AL, both the SVD and the DVD decreased. However, we did not find a significant decrease in the SVD of the PS group. This phenomenon has been similarly reported in a previous study (Nie et al., 2021). Another study hypothesized that impaired hemodynamics in myopic eyes is an early sign of a decrease in ocular blood flow in patients with pathological myopia (Benavente-Pérez et al., 2010). Therefore, we speculate that even if RT is reduced in eyes with PS, retinal blood flow density might have a compensatory role at a certain stage of myopia development. The decline in DVD might emerge earlier, while the change in the SVD was not significantly reduced until the microcirculation was completely out of compensation.

We also found that the foveal and parafoveal CCPA were significantly correlated with PS, which does not seem to have been reported to date. This indicated that microcirculatory abnormalities were closely correlated with the presence of PS. Localized bulging of the posterior pole of the fundus in the PS may lead to mechanical stretching, consequent thinning of the retina and choroid, straightening and thinning of the blood vessels, and consequently reducing CCPA. Decreased oxygen and blood supply may lead to tissue atrophy, which in turn may cause further localized bulging and a decrease in the adaptability of the blood supplement. However, the causality remains uncertain.

As SFST was found to be another factor significantly correlated with PS, we further explored the factors correlated with SFST. SFST was correlated with both age and SER in eyes with high myopia. With increasing age and exacerbation of refractive error, the SFST became lower in eyes with high myopia. Moreover, SFST was positively correlated with foveal DVD, RT, and CCPA. The lower the foveal DVD, RT, and CCPA, the lower the SFST. To the best of our knowledge, this is the first study to find that the thinning of SFST in PS was correlated with the abnormalities of macular microcirculation. Abnormalities in the microcirculation may cause pathological alterations of the scleral. Wu et al. (2018) indicated that hypoxia was an activator of extracellular matrix remodeling in the sclera during the progression of myopia. We speculated that the progression of myopia might lead to thinning of the choroid and abnormal microcirculation (Devarajan et al., 2020). In other words, lower CCPA and lower foveal DVD may cause decreased oxygen concentration levels. This could result in reduced nutrient and oxygen supply from the choroid to the neighboring avascular sclera. Scleral hypoxia may disrupt collagen fibrillogenesis and fibrolamellar reconstruction, resulting in progressive thinning of the sclera (Liu et al., 2016; Jonas et al., 2020). The thinner area had less resistance to the tangential force generated by the growth of the AL, which may have led to the development of PS. The histological and biomechanical properties of the sclera may play an important role in the occurrence of PS. However, these theories are still insufficient to fully explain the pathogenesis of PS in high myopic eyes, because the PS was also observed in some patients without high myopia (Xu et al., 2019; El Matri et al., 2020). Whether anti-hypoxic drugs can be applied to inhibit scleral thinning and control the development of myopia needs further study. Causality between PS and these alterations still needs to be corroborated by experimental studies in biology.

There are several limitations to the present study. Firstly, this was a cross-sectional study, and the sample size was relatively small. We excluded 16 eyes with poor imaging when we measured scleral thickness. Longitudinal follow-up observation with more samples may be able to discover the causality and precedence between PS and variables, as well as the risk factors for PS. Second, even after matching AL, all confounding factors for PS cannot be completely excluded. In addition, the application of SS-OCT for the diagnosis of PS has some limitations, as it was difficult to accurately measure the size of the macular area involved in PS and there was no classification of PS. A comparison between different types of PS would be beneficial to further reveal the differences between eyes with different morphologies of PS.

Myopia prevention and control have become urgent worldwide, which can be achieved through the prevention of PS. Future studies can combine 3D-MRI to identify the morphology of PS. Once the pathogenesis of PS is clarified, it will be possible to delay or reduce the visual impairment caused by pathological myopia. Since the presence of PS was strongly correlated with decreased microcirculation, whether the progression of myopia can be prevented by increasing the blood perfusion of the retina and choroid needs to be further investigated.

## Conclusion

PS was correlated with abnormalities in macular structures and microcirculation. SFST, CT, and CCPA were significantly correlated with PS. Interestingly, lower SFST in PS was correlated with abnormalities in choroidal thickness and microcirculation. Whether alterations in microcirculation can be the risk factor for PS needs to be further corroborated by longitudinal studies.

## Data Availability

The raw data supporting the conclusion of this article will be made available by the authors, without undue reservation
